# Supplementary material for: Assessing Reduction of Antibiotic Prescribing for Acute, Non-Complicated Infections in Primary Care in Germany: Multi-Step Outcome Evaluation in the Cluster-Randomized Trial ARena
Source: Antibiotics (Basel). 2021 Sep 24;10(10):1151. doi: 10.3390/antibiotics10101151 (PMC8532997; doi:10.3390/antibiotics10101151)
Supplement: Supplementary file 1 [file antibiotics-10-01151-s001.zip › antibiotics-1390974-Supplementary.pdf]

Supplementary Materials

Supplementary File S1:

Table S1: Distribution of medical specialty groups pre and post intervention.

**Intervention arm**	**I**		**II**		**III**		**Matched standard care**	
	**pre (n = 68)**	**post (n = 68)**	**pre (n = 57)**	**post (n = 57)**	**pre (n = 68)**	**post (n = 67)**	**pre (n = 190)**	**post (n = 190)**
Medical specialty group								
General Practice	53 (77.9%)	53 (77.9%)	43 (75.4%)	43 (75.4%)	50 (73.5%)	51 (76.1%)	144 (75.8%)	144 (75.8%)
Otolaryngology	6 (8.8%)	6 (8.8%)	7 (12.3%)	7 (12.3%)	6 (8.8%)	6 (9.0%)	17 (8.9%)	17 (8.9%)
Urology	5 (7.4%)	5 (7.4%)	2 (3.5%)	2 (3.5%)	4 (5.9%)	4 (6.0%)	13 (6.8%)	13 (6.0%)
Pediatrics	1 (1.5%)	1 (1.5%)	2 (3.5%)	2 (3.5%)	0 (0%)	0 (0%)	5 (2.6%)	5 (2.6%)
Gynecology	1 (1.5%)	1 (1.5%)	1 (1.8%)	1 (1.8%)	6 (8.8%)	5 (7.5%)	8 (4.2%)	8 (4.2%)
Other	2 (2.9%)	2 (2.9%)	2 (3.5%)	2 (3.5%)	2 (2.9%)	1 (1.5%)	3 (1.6%)	3 (1.6%)

Table S2: Patients with index disease—pre-/post-intervention and comparison to matched standard care.

		Intervention arm I		Intervention arm II		Intervention arm III		Matched standard care (no intervention)	
		**Pre (n = 9673)**	**Post (n = 10143)**	**Pre (n = 4583)**	**Post (n = 6730)**	**Pre (n = 3951)**	**Post (n = 5076)**	**Pre (n = 25.385)**	**Post (n = 25.144)**
Age	18-65	7537 (77.9%)	7869 (77.6%)	3497 (76.3%)	4684 (69.6%)	2972 (75.2%)	3910 (77.0%)	18,974 (74.7%)	18,960 (75.4%)
	<18	707 (7.3%)	578 (5.7%)	297 (6.5%)	757 (11.2%)	145 (3.7%)	143 (2.8%)	4,084 (16.1%)	4,210 (16.7%)
	>65	1429 (14.8%)	1696 (16.7%)	789 (17.2%)	1289 (19.2%)	834 (21.1%)	1023 (20.2%)	2,327 (9.2%)	1,974 (7.9%)
Gender	Male	4147 (42.9%)	4405 (43.4%)	1860 (40.6%)	2900 (43.1%)	1606 (40.6%)	2074 (40.9%)	11,688 (46.0%)	11,891 (47.3%)
	Female	5526 (57.1%)	5738 (56.6%)	2723 (59.4%)	3830 (56.9%)	2345 (59.4%)	3002 (59.1%)	13,697 (54.0%)	13,253 (52.7%)
Nationality	German	8962 (92.6%)	9115 (89.9%)	3693 (80.6%)	5425 (80.6%)	3325 (84.2%)	4144 (81.6%)	19.899 (78.4%)	18,681 (74.3%)
	Northern European	21 (0.2%)	26 (0.3%)	26 (0.6%)	39 (0.6%)	32 (0.8%)	37 (0.7%)	149 (0.6%)	181 (0.7%)
	Eastern European, Turkish, Arabian	596 (6.2%)	847 (8.4%)	607 (13.2%)	901 (13.4%)	427 (10.8%)	680 (13.4%)	3,995 (15.7%)	4,854 (19.3%)
	Other	44 (0.5%)	74 (0.7%)	81 (1.8%)	117 (1.7%)	38 (1.0%)	62 (1.2%)	476 (1.9%)	543 (2.2%)

		Intervention arm I		Intervention arm II		Intervention arm III		Matched standard care (no intervention)	
		Pre (n = 9673)	Post (n = 10143)	Pre (n = 4583)	Post (n = 6730)	Pre (n = 3951)	Post (n = 5076)	Pre (n = 25.385)	Post (n = 25.144)
	Southern European	50 (0.5%)	81 (0.8%)	176 (3.8%)	248 (3.7%)	129 (3.3%)	153 (3.0%)	866 (3.4%)	885 (3.5%)
Charlson Comorbid-ity Index	0	5621 (58.1%)	5734 (56.5%)	2462 (53.7%)	3858 (57.3%)	1976 (50.0%)	2604 (51.3%)	16,842 (66.3%)	17,412 (69.2%)
	1, 2	2761 (28.5%)	2926 (28.8%)	1445 (31.5%)	1945 (28.9%)	1276 (32.3%)	1609 (31.7%)	6,816 (26.9%)	6,259 (24.9%)
	3, 4	624 (6.5%)	781 (7.7%)	365 (8.0%)	453 (6.7%)	345 (8.7%)	492 (9.7%)	966 (3.8%)	830 (3.3%)
	>=5	667 (6.9%)	702 (6.9%)	311 (6.8%)	474 (7.0%)	354 (9.0%)	371 (7.3%)	761 (3.0%)	643 (2.6%)
Bronchitis		2931 (30.3%)	2442 (24.1%)	1231 (26.9%)	1569 (23.3%)	1343 (34.0%)	1457 (28.7%)	6,322 (24.9%)	5,796 (23.1%)
URTI		6301 (65.1%)	7620 (75.1%)	3037 (66.3%)	4916 (73.0%)	2720 (68.8%)	3719 (73.3%)	17,340 (68.3%)	17,663 (70.2%)
Sinusitis		968 (10.0%)	962 (9.5%)	453 (9.9%)	867 (12.9%)	485 (12.3%)	700 (13.8%)	1,712 (6.7%)	1,642 (6.5%)
Tonsillitis		559 (5.8%)	507 (5.0%)	327 (7.1%)	335 (5.0%)	166 (4.2%)	220 (4.3%)	1,753 (6.9%)	1,654 (6.6%)
Otitis Me-dia		459 (4.7%)	474 (4.7%)	270 (5.9%)	357 (5.3%)	139 (3.5%)	263 (5.2%)	1,826 (7.2%)	1,809 (7.2%)

Supplementary File S2:

Table S3: Percentage of cases with index disease receiving prescription for recommended antibiotics (pre-/post-intervention).

		**Intervention arm 1**		**Intervention arm 2**		**Intervention arm 3**		**Matched standard care (no intervention)**	
		**Pre**	**Post**	**Pre**	**Post**	**Pre**	**Post**	**Pre**	**Post**
Primary Outcome	Sample Size of endpoint population	9,673	10,143	4,583	6,730	3,951	5,076	25,385	25,144
	% primary outcome	31.8%	20.1%	28.8%	18.9%	36.3%	23.6%	33,9%	29,4%
SO1: Fluoroquinolones	Sample Size of endpoint population	3,119	2,079	1,391	1,347	1,488	1,249	8,885	7,655
	% Fluoroquinolones	11.3%	7.3%	7.7%	4.5%	9.1%	7.1%	9.1%	7.5%
SO2: Recommended AB acute URTI	Sample Size of endpoint population	1,541	1,206	626	613	827	734	4,311	3,637
	% Recommended AB acute URTI	18.7%	25.5%	18.7%	32.6%	18%	25.3%	16,7%	18,7%
SO3: Recommended AB Bronchitis	Sample Size of endpoint population	1,310	727	551	463	681	503	3,115	2,684
	% Recommended AB Bronchitis	26.6%	32.5%	16.7%	28.7%	21.3%	30.6%	19,0%	23,2%
SO4: Recommended AB Sinusitis	Sample Size of endpoint population	411	300	122	196	215	201	904	768
	% Recommended AB Sinusitis	18.7%	23.0%	25.4%	28.1%	26.5%	31.8%	16.4%	18.8%
SO5: Recommended AB Tonsillitis	Sample Size of endpoint population	403	355	276	274	150	171	1,511	1,388
	% Recommended AB Tonsillitis	22.8%	33.5%	17.4%	20.8%	10.7%	25.7%	25.0%	28.9%
SO6: Recommended AB Otitis Media	Sample Size of endpoint population	137	145	92	104	54	96	774	711
	% Recommended AB Otitis Media	27.7%	37.9%	27.2%	41.3%	33.3%	25.0%	23.6%	29.3%

SO = Secondary Outcome; AB = antibiotics; URTI = upper respiratory tract infection

Table S4: Comparison of intervention arms to standard care regarding secondary outcomes adjusted for additional covariates.

Secondary Outcome		OR	95% CI of OR		p-Wert
			lower	upper	
acute bronchitis with a prescription for recommended antibiotics	Intervention arm I vs standard care	1.649	1.452	1.872	<0.001
	Intervention arm II vs standard care	0.995	0.837	1.18	0.957
	Intervention arm III vs standard care	1.391	1.196	1.615	<0.001
	post vs pre	1.443	1.312	1.587	<0.001
	Age >65 vs 18-65	0.996	0.992	0.999	0.017
	Female vs male	0.933	0.848	1.027	0.159
	CCI 1,2 vs 0	0.666	0.600	0.739	<0.001
	CCI 3,4 vs 0	0.641	0.525	0.778	<0.001
	CCI >=5 vs 0	0.711	0.572	0.879	0.002
	Northern Europe vs GER	0.766	0.347	1.514	0.473
	Eastern Europe, Turkey, Arab. vs GER	1.246	1.082	1.432	0.002
	Southern Europe vs GER	1.148	0.76	1.694	0.497
	Other nationality vs GER	1.146	0.861	1.508	0.341
	urban vs rural	1.257	1.126	1.403	<0.001
sinusitis with a prescription for recommended antibiotics	Intervention arm I vs standard care	1.335	1.055	1.685	0.016
	Intervention arm II vs standard care	1.37	1.011	1.848	0.04
	Intervention arm III vs standard care	2.218	1.704	2.878	<0.001
	post vs pre	1.248	1.046	1.49	0.014
	Age >65 vs 18-65	0.87	0.648	1.158	0.347
	Female vs male	0.904	0.753	1.087	0.28
	CCI 1,2 vs 0	0.97	0.789	1.189	0.771
	CCI 3,4 vs 0	1.065	0.71	1.569	0.754
	CCI >=5 vs 0	0.843	0.516	1.343	0.484
	Northern Europe vs GER	1.123	0.351	3.098	0.831
	Eastern Europe, Turkey, Arab. vs GER	0.768	0.57	1.022	0.076
	Southern Europe vs GER	2.263	1.248	4.007	0.006
	Other nationality vs GER	0.631	0.286	1.247	0.216
	urban vs rural	1.508	1.224	1.855	<0.001
tonsillitis with a prescription for recommended antibiotics	Intervention arm I vs standard care	1.154	0.948	1.401	0.152
	Intervention arm II vs standard care	0.683	0.536	0.863	0.002
	Intervention arm III vs standard care	0.682	0.501	0.914	0.012
	post vs pre	1.335	1.166	1.529	<0.001
	Age <18 vs 18-65	1.15	0.976	1.351	0.093
	Age >65 vs 18-65	0.756	0.487	1.138	0.194
	Female vs male	1.017	0.886	1.168	0.807
	CCI 1,2 vs 0	0.735	0.606	0.887	0.001
	CCI 3,4 vs 0	0.518	0.284	0.883	0.022
	CCI >=5 vs 0	0.48	0.236	0.893	0.029
	Northern Europe vs GER	1.258	0.563	2.627	0.555
	Eastern Europe, Turkey, Arab. vs GER	0.985	0.823	1.176	0.869

	Other nationality vs GER	0.921	0.442	1.775	0.814
	Southern Europe vs GER	0.789	0.515	1.174	0.257
	urban vs rural	0.982	0.843	1.144	0.814
acute URTI with a prescription for recommended antibiotics	Intervention arm I vs standard care	1.598	1.421	1.796	<0.001
	Intervention arm II vs standard care	1.454	1.253	1.685	<0.001
	Intervention arm III vs standard care	1.552	1.349	1.782	<0.001
	post vs pre	1.357	1.245	1.479	<0.001
	Age <18 vs 18-65	1.661	1.438	1.913	<0.001
	Age >65 vs 18-65	0.808	0.698	0.934	0.004
	Female vs male	0.927	0.849	1.012	0.089
	CCI 1,2 vs 0	0.909	0.823	1.003	0.058
	CCI 3,4 vs 0	0.848	0.689	1.037	0.114
	CCI >=5 vs 0	0.913	0.733	1.131	0.411
	Northern Europe vs GER	0.875	0.442	1.599	0.682
	Eastern Europe, Turkey, Arab. vs GER	0.954	0.837	1.086	0.481
	Southern Europe vs GER	1.12	0.777	1.582	0.53
	Other nationality vs GER	0.707	0.527	0.932	0.017
	urban vs rural	1.536	1.392	1.696	<0.001
otitis media with a prescription for recommended antibiotics	Intervention arm I vs standard care	2.147	1.577	2.917	<0.001
	Intervention arm II vs standard care	1.445	1.026	2.023	0.033
	Intervention arm III vs standard care	1.733	1.149	2.581	0.008
	post vs pre	1.376	1.132	1.675	0.001
	Age <18 vs 18-65	1.855	1.479	2.328	<0.001
	Age >65 vs 18-65	1.011	0.662	1.522	0.958
	Female vs male	1.099	0.9	1.343	0.356
	CCI 1,2 vs 0	0.908	0.704	1.166	0.454
	CCI 3,4 vs 0	0.812	0.462	1.383	0.455
	CCI >=5 vs 0	0.388	0.163	0.815	0.019
	Northern Europe vs GER	1.072	0.285	3.341	0.909
	Eastern Europe, Turkey, Arab. vs GER	1.144	0.866	1.504	0.338
	Southern Europe vs GER	1.545	0.57	3.844	0.364
	Other nationality vs GER	1.258	0.724	2.131	0.402
	urban vs rural	1.762	1.413	2.203	<0.001
quinolone prescription	Intervention arm I vs standard care	0.921	0.821	1.031	0.156
	Intervention arm II vs standard care	0.713	0.597	0.848	<0.001
	Intervention arm III vs standard care	0.72	0.618	0.837	<0.001
	post vs pre	0.74	0.677	0.809	<0.001
	Age <18 vs 18-65	8.865	5.954	13.969	<0.001

Age >65 vs 18-65	11.687	7.724	18.647	<0.001
Female vs male	0.999	0.915	1.092	0.989
CCI 1,2 vs 0	1.588	1.441	1.751	<0.001
CCI 3,4 vs 0	1.902	1.611	2.239	<0.001
CCI >=5 vs 0	2.029	1.698	2.416	<0.001
Northern Europe vs GER	0.778	0.365	1.452	0.47
Eastern Europe, Turkey, Arab. vs GER	0.902	0.78	1.038	0.155
Southern Europe vs GER	0.849	0.543	1.266	0.448
Other vs GER	1.152	0.881	1.483	0.287
urban vs rural	0.742	0.671	0.821	<0.001

Supplementary File S3:

Table S5: Diagnoses Primary Outcome.

Diagnoses	ICD-10 Code
Upper Respiratory Infections	J00; J02.0; J02.8, J02.9; J04; J06; J10.1; J11.1
Bronchitis	J20; J21.0; J21.1, J21.8; J21.9; J22; J40
Tonsillitis	J03.9
Sinusitis	J01
Otitis Media	H65.0; H65.1; H65.9; H66.0; H66.4; H66.9

Table S6: Disregarded Diagnoses *

A00 bis A79.9	infectious colon diseases; Tuberculosis; specific bacterial zoonoses; other bacterial diseases; sexually transmitted diseases; other spirochetes diseases; other chlamydia-induced diseases; Rickettsioses
E84	Cystic fibrosis
J44.0, J44.1	Other chronic obstructive lung diseases

N10	Acute tubulo-interstitial nephritis
N12	Nephritis not acute or interstitial
N30	Cystitis
N39.0	Other urinary tract infections
N41	Inflammatory prostate diseases
O	Pregnancy, birth, puerperium

* Cases with disregarded diagnoses in an observed quarter are not considered for analysis.

Table S7: Currently recommended and alternative Antibiotics.

	**Recommended Antibiotics**	**Alternative Antibiotics**
Upper Respiratory Infections	J01CA04 (Amoxicillin)	-
Bronchitis	J01CA04 (Amoxicillin)	J01AA02 (Doxycyclin); J01CE02 (Phenoxymethylpenicillin); J01FA (Macrolides)
Tonsillitis	J01CE02 (Phenoxymethylpeni-cillin)	J01DB (Cephalosporins); J01FA01(Erythromycin)
Sinusitis	J01CA04 (Amoxicillin)	J01DC (Cephalosporins 2nd Gen); J01AA02 (Doxycyclin); J01EE01 (Sulfamethoxazol and Trimethoprim); J01CR02 (Amoxicillin and beta-lac-tamase-inhibitors); J01CR22 (Amoxicillin and Clavulanic acid)
Otitis Media	J01CA04 (Amoxicillin)	J01DC (Cephalosporins 2nd Gen); J01FA01 (Erythromycin); J01CR02 (Amoxicillin and beta-lactamase-inhibitors); J01CR22 (Amoxicillin and Clavulanic acid)
